# Identifying Predictors of COVID-19 Mortality Using Machine Learning

**DOI:** 10.3390/life12040547

**Published:** 2022-04-06

**Authors:** Tsz-Kin Wan, Rui-Xuan Huang, Thomas Wetere Tulu, Jun-Dong Liu, Asmir Vodencarevic, Chi-Wah Wong, Kei-Hang Katie Chan

**Affiliations:** 1 Department of Electrical Engineering, City University of Hong Kong, Hong Kong, China; tszkinwan2-c@my.cityu.edu.hk (T.-K.W.); rxhuang4-c@my.cityu.edu.hk (R.-X.H.); 2 Department of Biomedical Sciences, City University of Hong Kong, Hong Kong, China; thomas.wetere@cityu.edu.hk (T.W.T.); jdliu4-c@my.cityu.edu.hk (J.-D.L.); 3 Computational Data Science Program, Addis Ababa University, Addis Ababa 1176, Ethiopia; 4 Novartis Oncology, Novartis Pharma GmbH, 90429 Nuremberg, Germany; asmir.vodencarevic@novartis.com; 5 Department of Applied AI and Data Science, City of Hope, Duarte, CA 91010, USA; alecwong@coh.org; 6 Department of Epidemiology and Center for Global Cardiometabolic Health, School of Public Health, Brown University, Providence, RI 02912, USA

**Keywords:** COVID-19 mortality, COVID-19, prediction model, machine learning model, COVID-19, mortality predictors

## Abstract

(1) Background: Coronavirus disease 2019 (COVID-19) is a dominant, rapidly spreading respiratory disease. However, the factors influencing COVID-19 mortality still have not been confirmed. The pathogenesis of COVID-19 is unknown, and relevant mortality predictors are lacking. This study aimed to investigate COVID-19 mortality in patients with pre-existing health conditions and to examine the association between COVID-19 mortality and other morbidities. (2) Methods: De-identified data from 113,882, including 14,877 COVID-19 patients, were collected from the UK Biobank. Different types of data, such as disease history and lifestyle factors, from the COVID-19 patients, were input into the following three machine learning models: Deep Neural Networks (DNN), Random Forest Classifier (RF), eXtreme Gradient Boosting classifier (XGB) and Support Vector Machine (SVM). The Area under the Curve (AUC) was used to measure the experiment result as a performance metric. (3) Results: Data from 14,876 COVID-19 patients were input into the machine learning model for risk-level mortality prediction, with the predicted risk level ranging from 0 to 1. Of the three models used in the experiment, the RF model achieved the best result, with an AUC value of 0.86 (95% CI 0.84–0.88). (4) Conclusions: A risk-level prediction model for COVID-19 mortality was developed. Age, lifestyle, illness, income, and family disease history were identified as important predictors of COVID-19 mortality. The identified factors were related to COVID-19 mortality.

## 1. Introduction

Coronavirus disease 2019 (COVID-19) is a dominant respiratory disease [1] that has circulated globally from 2020 and was characterized as a pandemic on 11 March 2020 [2]. The COVID-19 pandemic has caused a severe global health threat and increased the processing burden of worldwide healthcare systems [3]. As of 25 December 2021, there were 283 million confirmed cases and 5.41 million confirmed deaths due to COVID-19 across more than 237 countries, with a global mortality of 1.9% and a steep daily increase in the number of cases [4]. COVID-19 is related to severe acute respiratory syndrome [5].

Age is one of the risk factors well-known for severe COVID-19 [6], but age did not show the typical U-shaped risk curve of COVID-19, which is different from other respiratory diseases [7]. However, the factors influencing COVID-19 mortality still have not been confirmed. In addition, the pathogens of COVID-19 are unknown [8], and relevant mortality predictors are lacking. However, a recent study showed that biomarkers may help identify relevant clinical outcomes [9]. COVID-19 survivors may experience persistent pulmonary disease. A study from China investigating the long-term sequelae of COVID-19 suggested that COVID-19 causes chronic damage to the cardiovascular system [10]. Identifying potential risk profiles might help in the early identification of patients with a poor prognosis. The pathogenesis of COVID-19 is unknown, and predictors of mortality due to COVID-19 are lacking. However, a recent study showed that patient characteristics might help identify relevant clinical outcomes [11]. Machine learning (ML) can analyze various variables in a biological compartment and identify patterns associated with specific disorders [12].

In this study, we used machine learning models and data mining to identify the relationship between various patient characteristics and COVID-19. We also aimed to investigate COVID-19 mortality in patients with pre-existing health conditions and examine the association between COVID-19 mortality and other morbidities, such as diabetes, cardiovascular cancers, and neurodegenerative diseases.

## 2. Materials and Methods

De-identified data from 113,882 individuals, including 14,877 COVID-19 patients collected from the UK Biobank, were used in this study. The UK Biobank is a major biomedical database and research resource that contains different types of data related to COVID-19. Our dataset included 17,954 features and a target variable. Of the COVID-19 patients, 799 patients died from the disease.

Four machine learning (ML) models: the Deep Neural Networks model (DNN), Random Forest Classifier (RF), XGBoost classifier (XGB) and Support Vector Machine (SVM), were used in this study. These four models were used to predict the mortality risk level of COVID-19 and were chosen because the DNN model provides the best performance for deep learning when the size of dataset is large and advanced in handle complicate relationship between input features and target feature. There are three disadvantages of the DNN model. Firstly, DNN model may need more than ten times the training time to achieve the best performance. Secondly, the DNN model requires very large amount of data to perform better than other ML models. The DNN model also has no standard theory for choosing parameters and training method, while the RF and XGB models provide a suitable algorithm to produce a prediction model while reducing the risk of overfitting. Although the RF and XGB models share a similar structure, the RF model on complex problems is usually poorer than the XGB trees; and the SVM model often provides a fair result compared with other machine learning models; it is a simplified model with the fast run time. SVM model may be disadvantaged when the relationship between input features and target feature are complicated and unapparent.

The ML models were built using Python with the integrated development environment provided by PyCharm 2021.2.2 (runtime version: 11.0.12 + 7-b1504.28 amd64), using an OpenJDK 64-Bit Server VM (JDK version: JetBrains s.r.o., Prague, Czech Republic), with Anaconda3 and Anaconda Navigator 2.0.4 as the project interpreters. Numpy [13] and Pandas [14] were used to process arrays and matrices as a dataframe and to process read/write data and operating data. To build the ML models, Sklearn [15] provided the application programming interface (API) of the ensemble classifier for the RF classifier, XGB and SVM and the API for data preprocessing. Sklearn also provided the ML model platform, and Keras [16] provided implementations of neural networks based on Tensorflow and Theano.

Table 1 shows the two fundamental data statistics in the UK Biobank original dataset, including all patients and patients who died due to COVID-19.

### 2.1. Features Merging

For features merging, there were 17,954 features in the raw dataset including 13,496 non-empty features from the UK Biobank. Some of the features could be merged into a new single feature based on the same feature semantic, as shown by the examples in Table 2.

Table 2 shows an example of features represented the ‘weight method’ but using different UDI from 21-0.0 to 21-3.0. Therefore, those features could be merged directly using the participant ID, as they had the same units and meaning. After merging the features by UDI grouping, the number of features decreased to 3442. After the primary merge by UDI grouping, some features represented the same measurement and were divided into different UDI groupings. Table 3 shows one of these examples, in which features that were assigned to UDI 94 and UDI 4079 groupings could be merged into one feature.

### 2.2. Scaling

In data scaling, feature values were scaled to the interval [−1,1] and outliers in the ML and neural network models were removed to avoid domination by extremely large values and to create a similar range and difference, by min-max normalization (Equation (1)) [17]. Outliers that were greater than three standard deviations from the mean were removed.

*Z* = *min* + ((*max* − *min*) ∗ (*unscaledData* − *min*)/(*max* − *min*)) (1)

### 2.3. Data Preprocessing and Feature Selection

Primary feature selection involved the elimination of missing data. The threshold of missing data was 30%, and 296 features were eliminated by primary feature selection. The second filter involved removal of irrelevant data. For example, variables such as ‘Blood pressure device ID’ and ‘Height measure device ID’ are not relevant predictors.

The train-test ratio of splitting data was 80:20 and an iterative imputation method MissForest [18] was applied to replace the missing value in the training set.

Regression input perturbation ranking [19] was used for primary feature selection using the K-best algorithm through a chi-squared distribution (Equation (2)) [20]. The importance and collinear nature of the features were used in the selection process. A total of 229 features with zero importance were identified after one-hot encoding, 240 features were found to have a cumulative importance of 0.95 after one-hot encoding, and 469 out of 540 features were identified for removal after one-hot encoding. There were 71 of input as listed in Table A1.

(2) $$\frac{1}{2}*\;x^{2}=\overset{n}{\underset{i=1}{\sum }}\left(\frac{{\left(x_{i}-\upsilon_{i}\right)}^{2}}{2\sigma_{i}^{2}}\right)$$

### 2.4. Model

#### 2.4.1. Deep Neural Network

The DNN model used four fully connected hidden layers, one input layer and one output layer. The first hidden layer had 268 neurons, nearly double the number of input features. The number of neurons in the higher layer is decreased layer by layer decide by grid search hyperparameter tuning tools. RandomNormal was used as the initializer, the activation of hidden layers was performed using Relu, and Adadelta was used as the optimiser. Early stopping was used during the training process to monitor validation loss.

#### 2.4.2. Random Forest Classifier

The RF model used the ‘Gini’ impurity metric (mean decrease in impurity) to calculate feature importance one by one individually. The GridSearchCV in RF model was applied to adjust the value of parameters. The number of estimators was set at 279, and the maximum depth of the trees was set at 5. The number of features to consider when looking for the best split is the square of the number of input features; the minimum number of samples and leaf required to split an internal node was set at 1 and 4, respectively.

#### 2.4.3. XGBoost Classifier

The XGB model applied the GridSearchCV impurity metric to adjust the value of parameters. The number of estimators was set at 200, maximum tree depth was set at 8, and minimum child weight set at 1.

#### 2.4.4. Linear SVM

The linear SVM model was mainly used to address the problem of dividing the results by a linear equation. The linear SVM model performs well when the number of features is large. Moreover, the speed of training is faster for linear SVM models than for other SVM models. Under normal circumstances, the linear SVM model has acceptable performance compared with neural networks.

### 2.5. Imbalanced Classification

The synthetic Minority Oversampling Technique (SMOTE) was applied for each ML model. By oversampling the minority class for ML models effectively learn the decision boundary.

### 2.6. Output Result

The target variable was binary (patient death yes or no). Machine learning models were applied to predict the probability of death, ranging from 0 to 1, with values close to 0 indicating a low risk, and those close to 1 indicating a high risk. Table 4 shows the training information of each ML model.

## 3. Results

We used DNN, RF, XGB, and linear SVM models for COVID-19 mortality prediction. Of the four ML models tested, the RF model provided the best results. The output of the models was a continuous number from 0 to 1, representing the probability of COVID-19 mortality by 5-fold cross-validation. Table 5 shows the AUC values for the risk level results from the DNN, RF, XGB, and linear SVM models.

**Figure 1:**
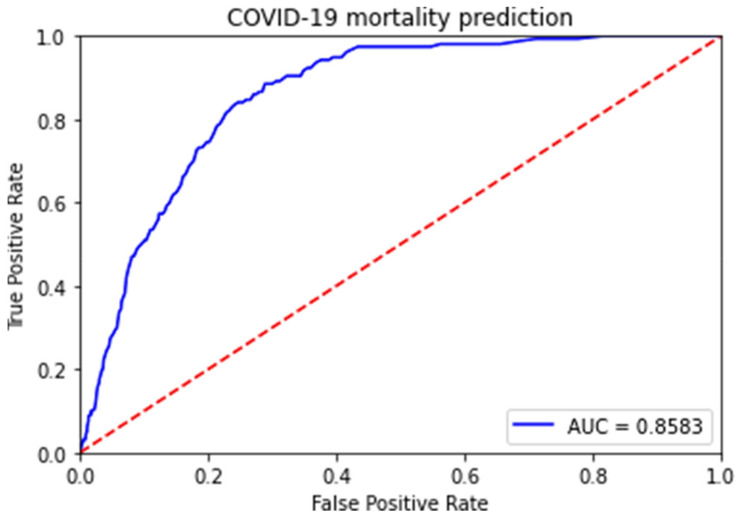
Receiver operating characteristic curve of the RF model.

Figure 1 shows the AUC value of the prediction result of the RF classifier model. The RF classifier model showed the best results of the three machine learning models. The mortality risk of COVID-19 was found to be 0.86 (95% CI: 0.84–0.88).

**Figure 2:**
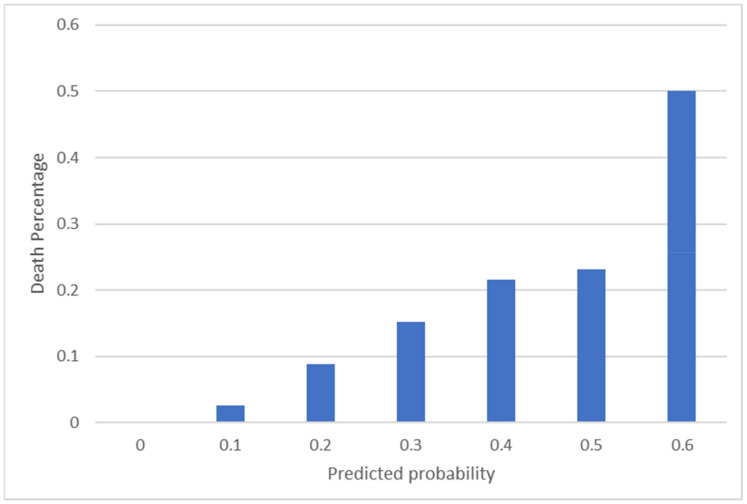
Predicted results and corresponding mortality rates.

Table 6 and Figure 2 show the predicted results and corresponding mortality rates. For example, when a patient had a predicted mortality probability of [0, 0.1), the survival rate was 99.8%, but when a patient had a predicted mortality probability of [0.6, 0.7), the survival rate was only 50%.

For binary classification, a novel threshold-based and k-means clustering method [21] was used to convert the regression results to binary classification results. The best results generated by the RF model gave an AUC value of 0.79.

**Figure 3:**
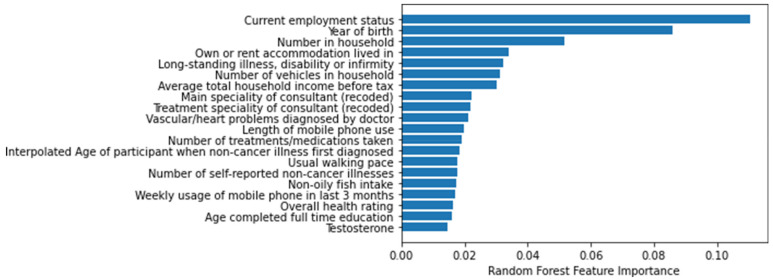
Top 20 important features for the RF model.

Figure 3 lists the 20 most important features of the RF model. These features were related to age, lifestyle, illness, income, and family history.

**Figure 4:**
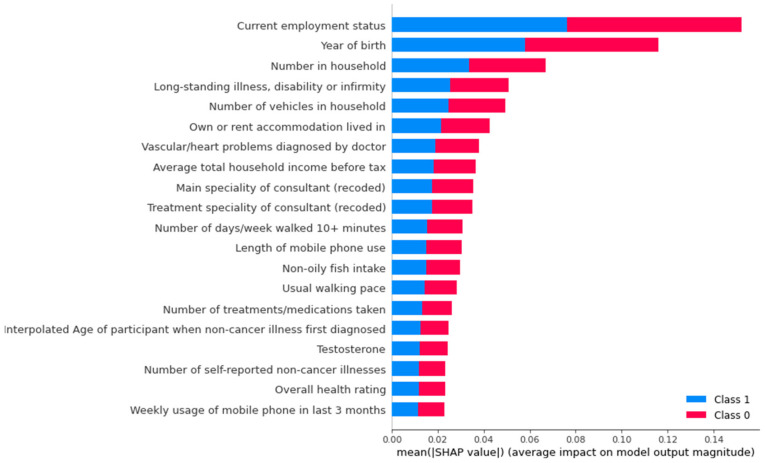
SHapley Additive exPlanations.

**Figure 5:**
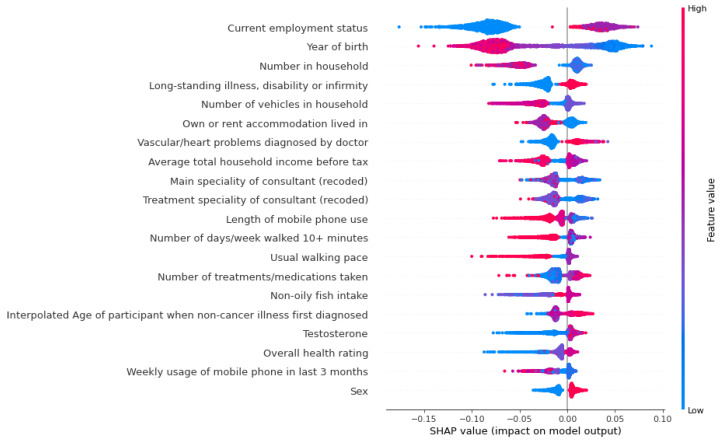
SHapley Additive exPlanations.

Figure 4 and Figure 5 show the SHAP value break down related to the impact of top 20 features based on magnitude of feature attributions. Positive or negative SHAP values indicate the effect of COVID-19 mortality for top 20 features.

## 4. Discussion

A risk level prediction model for COVID-19 mortality was developed in this study using data from the UK Biobank. We used a risk level to predict COVID-19 related mortality rather than a binary classification prediction because a risk level can be used to easily identify patients with a poor prognosis earlier by analyzing potential risk factors.

Table 7 shows several scoring systems to estimate the early risk of COVID-19, including the International Severe Acute Respiratory Infection Consortium Clinical Characterization Protocol-Coronavirus Clinical Characterization Consortium (ISARIC-4C) score, quick COVID-19 Severity Index (qCSI), National Early Warning Score 2 (NEWS2) and CURB-65 (confusion, uremia, respiratory rate, BP, age 65 years).

Compared with developed scoring systems, the RF model in this study achieve the best performance of COVID-19 mortality risk prediction.

Considering the time limit for running the model, the DNN model used a single hidden layer, the grid search hyperparameter tuning tools used for RF model estimators was set at only few options, and the maximum depth option of the trees was set at 1 to 5. Thus, the parameters of the model may not be fully optimized and although the results showed an AUC value of 0.86 (95% CI:0.84–0.88), a higher AUC value may have been possible. Another limitation of this study is that because there were more than a thousand features in the original dataset, many of the features were similar. We may not have been able to eliminate all of the related data because the correlations between them were lower than the threshold. For example, the features ‘year of birth’ and ‘mother still lives’ should be related under normal circumstances. The raw dataset also contains variables only at the individual level. Area-level data such as temperature, and income may increase the performance of prediction models [26], but were not provided in the detailed information of each patient in the original dataset.

For COVID-19 patients, age represented the highest risk, as more than five out of the 20 most important features were related to age of the patient. Income, lifestyle, disease history, and family disease history were also important features for COVID-19 patients. This study defined the important features related to COVID-19 mortality, and may provide an objective and quantitative risk model for clinical care.

## 5. Conclusions

This study found a significant relationship between specific patient characteristics and the risk of COVID-19-related death. Age, income/personal property, long-standing illness, disability, and heart disorders were important factors affecting COVID-19 mortality. Some unique features, such as ‘length of mobile phone use’ and ‘non-oily fish intake’, were relevant for predicting COVID-19 mortality but have not previously been reported. Alcohol intake showed no associated COVID-19 mortality in the prediction, which is inconsistent with what may be theoretically expected [27]. This study identified some patient characteristics that are not easily obtained but showed a relationship with COVID-19 mortality.

Future studies will aim to collect more laboratory testing data of confirmed COVID-19 cases and collect more detail information of patients for analysis at the area-level to increase the performance of the prediction models.

## Figures and Tables

**Table 1:** Basic characteristics of the UK Biobank study participants showed the mean and its one standard deviation or percentage and actual number of patients basic characteristics.

Basic UK Biobank Data Characteristics	Statistics (All Data, *n* = 14,877)	Statistic (Death Due to COVID-19, *n* = 799)
Age	66.5 (57.8, 75.1)	75.8 (55.9, 90.0)
Death	5.37% (*n* = 799)	N/A
Male gender	52.8%	34.2%
Height	168.6 (159.4, 177.8)	168.8(159.6, 178.0)
Weight	80.1 (64.4, 95.8)	84.0(67.3, 100.8)
Body mass index	28.0 (23.5, 32.6)	29.3(24.3, 34.4)
Current tobacco smoking	7.9% (*n*= 1176)	10.6% (*n* = 85)
Vascular/heart problems diagnosed by doctor	23.2% (*n*= 3451)	37.7% (*n* = 302)
Blood clot, deep-vein thrombosis, bronchitis, emphysema, asthma, rhinitis, eczema, or allergy diagnosed by a doctor	16.5% (*n* =2456)	22.2% (*n* = 177)
Other serious medical condition/disability diagnosed by a doctor	19.0% (*n* = 2833)	33.3% (*n* = 266)
Long-standing illness, disability or infirmity	33.5% (*n* = 4983)	57.4 (*n* = 459)
Alcohol consumption	95.9% (*n* =14,272)	93.3% (*n* = 746)

**Table 2:** Example of features representing the same meaning under the same UDI.

UDI ^1^	Data Size	Description
21–0.0	500,790	
21–1.0	20,334	Weight
21–2.0	46,439	method
21–3.0	2729	

^1^ UDI—the Unique Data Identifier for an item of data within the UK Biobank repository.

**Table 3:** Examples of features representing the same meaning in different UDI grouping.

UDI	Description
94	Diastolic blood pressure, manual reading
4079	Diastolic blood pressure, automated reading

**Table 4:** Data distribution.

Training and Prediction Round	50 Times
Prediction type	Regression
Total number of data point	In the DNN model: Training on 9521 samples (before SMOTE), validation on 2380 samples, and testing on 2975 samples In the RF, XGB and SVM models: Training on 11,901 samples (before SMOTE) and, testing on 2975 samples

**Table 5:** Regression results from different models obtained on the testing data.

Model	Result (AUC)
DNN	0.84 (95% CI: 0.81–0.85)
RF	0.86 (95% CI: 0.84–0.88)
Linear SVM	0.81 (95% CI: 0.79–0.83)
XGB	0.83 (95% CI: 0.82–0.86)

**Table 6:** Predicted results and corresponding mortality rates.

Predicted Probability %	Number of Predicted Patients	Number of Deaths	Mortality Rate %
[0,10)	1335.0	3.0	0.225
[10,20)	664.0	17.0	2.56
[20,30)	440.0	39.0	8.86
[30,40)	303.0	46.0	15.18
[40,50)	190.0	41.0	21.58
[50,60)	39.0	9.0	23.08
[60,70)	4.0	2.0	50
[70,80)	0.0	0.0	NaN
[80,90)	0.0	0.0	NaN
[90,100)	0.0	0.0	NaN

**Table 7:** Comparison of performance with developed scoring systems.

Model	AUC
RF model (This study)	0.863 (95% CI: 0.842–0.881)
NEWS2 [22]	0.790 (95% CI: 0.643–0.937)
CURB-65 [23]	0.81 (95% CI: 0.71–0.91)
ISARIC-4C [24]	0.79 (95% CI: 0.78–0.79)
qCSI [25]	0.81 (95% CI: 0.73–0.89)

## Data Availability

This research was conducted using data from UK Biobank, a major biomedical database (www.ukbiobank.ac.uk (accessed on 12 December 2021), project number: 45788).

## Appendix A

**Table A1:** Input features for models.

Feature Name			
Able to confide	Age at recruitment	Age completed full time education	Age first had sexual intercourse
Age when attended assessment centre	Alanine aminotransferase	Albumin	Alkaline phosphatase
Arm fat-free mass (left)	Arm fat-free mass (right)	Arm predicted mass (left)	Arm predicted mass (right)
Aspartate aminotransferase	Average total household income before tax	Birth weight known	Body mass index (BMI)
Body mass index (BMI)	Bread intake	Breastfed as a baby	Carer support indicators
Chest pain or discomfort	Cholesterol	Cooked vegetable intake	C-reactive protein
Creatinine	Current employment status	Cystatin C	Daytime dozing/sleeping (narcolepsy)
Direct bilirubin	Dried fruit intake	Eosinophill count	Ever had bowel cancer screening
Falls in the last year	Father still alive	Forced expiratory volume in 1-s (FEV1)	Forced expiratory volume in 1-s (FEV1) Z-score
Forced vital capacity (FVC)	Forced vital capacity (FVC) Z-score	Gamma glutamyltransferase	Genetic sex
Glucose	Glycated haemoglobin (HbA1c)	Haematocrit percentage	Haemoglobin concentration
HDL cholesterol	Hearing difficulty/problems	Hearing difficulty/problems with background noise	High light scatter reticulocyte count
High light scatter reticulocyte percentage	Housing score (England)	IGF-1	Illnesses of siblings
Immature reticulocyte fraction	Impedance of arm (left)	Impedance of arm (right)	Impedance of leg (left)
Impedance of leg (right)	Impedance of whole body	Intended management of patient (polymorphic)	Intended management of patient (recoded)
Interpolated Age of participant when non-cancer illness first	diagnosed	Interpolated Age of participant when operation took place	Interpolated Year when operation took place
IPAQ activity group	LDL direct	Leg fat-free mass (left)	Leg fat-free mass (right)
Leg predicted mass (left)	Leg predicted mass (right)	Length of mobile phone use	
